# Characterizing virtual community exercise programs for people with mobility limitations: a scoping review

**DOI:** 10.1016/j.jesf.2026.200477

**Published:** 2026-04-25

**Authors:** Renato Barbosa dos Santos, Jing Lin, Anchal Badwal, Hardeep Singh, Susan B. Jaglal, Chavon Niles, Nancy M. Salbach

**Affiliations:** a Rehabilitation Sciences Institute, Temerty Faculty of Medicine, University of Toronto, 160-500 University Ave, Toronto, ON, M5G 1V7, Canada; b Department of Physical Therapy, Temerty Faculty of Medicine, University of Toronto, 160-500 University Ave, Toronto, ON, M5G 1V7, Canada; c Department of Occupational Science & Occupational Therapy, Temerty Faculty of Medicine, University of Toronto, 500 University Ave, Toronto, ON, M5G 1V7, Canada; d KITE Research Institute, Toronto Rehabilitation Institute - University Health Network, 550 University Ave, Toronto, ON, M5G 2A2, Canada

## Abstract

Virtual delivery of exercise programs can overcome barriers associated with accessing in-person programs for people with mobility limitations. Despite the growing number of virtual programs, several gaps remain, including how these programs are delivered and the specific strategies used to engage participants safely. We aimed to describe the intervention and methodological characteristics of virtual community exercise programs for adults with mobility limitations. We conducted a scoping review of virtual community exercise programs for adults with health conditions associated with mobility limitations. Data were summarized descriptively, and outcomes were classified using the International Classification of Functioning, Disability and Health. A total of 36 studies were included. Pre-post studies (39%) and randomized trials (28%) were the most common study designs. Studies frequently targeted individuals with Parkinson's disease (31%), multiple sclerosis (11%), and stroke (11%). Programs were commonly group-based (44%), 2 classes/week (47%) lasting 60 min (63%), for 8 weeks (33%). Interventions frequently included strength (44%), aerobic (31%), and balance training (19%), and synchronous delivery (80%) by physiotherapists (39%) using Zoom™ (68%). Safety strategies included guidance for space and technology setup (19%), and pre-session safety checks (11%). A wide range of outcomes were evaluated, most commonly health-related quality of life (44%) and physical function (28%). This scoping review shows that virtual community exercise programs for adults with mobility limitations primarily involved physiotherapist-led, 60-min group sessions delivered via Zoom™, focusing on a combination of strength and aerobic training, and emerging mind–body modalities, and high attendance. Findings highlight the need for larger, well-powered trials with standardized outcomes to support scalability and real-world implementation.

## 1 Introduction

Mobility limitations refer to difficulties changing or maintaining body position (i.e., static and dynamic balance), walking, or moving around.^1^ These limitations are highly prevalent worldwide,^2^ and often associated with health conditions like stroke,^3^ osteoarthritis (OA),^4^ multiple sclerosis (MS),^5^ Parkinson's disease (PD),^6^ spinal cord injury,^7^ cognitive impairment (e.g., due to Alzheimer's disease or dementia),^8^ and frailty.^9^ For people with mobility limitations, exercise interventions that focus on functional activities like sit-to-stand, stepping, and walking, have shown potential for improving balance, walking capacity, and overall mobility (i.e., moving around) for people with mobility limitations.^10^, ^11^, ^12^, ^13^ Despite the benefits of exercise programs,^14^, ^15^, ^16^, ^17^, ^18^, ^19^, ^20^ people with mobility limitations face barriers to attending in-person exercise programs. Fear of adverse events, lack of motivation or time, along with barriers related to transportation, inaccessible building, inclement weather, and pandemic-related program closures, make it challenging to engage in in-person exercise programs.^21^, ^22^, ^23^, ^24^ In addition, for caregivers the need to provide transportation or supervision can extend caregiving time and contribute to caregiver burden.^14^^,^^25^^,^^26^

Virtual delivery of exercise programs can overcome barriers associated with accessing in-person programs.^27^ Evidence shows that rehabilitation programs delivered through technology have similar effects to in-person delivery on everyday activities and mobility outcomes.^28^^,^^29^ However, because formal rehabilitation is time-limited and many individuals lack continued support,^30^ ongoing access to exercise programs outside of traditional healthcare settings is essential, and virtual exercise programs offer a feasible way to provide this continuity in physical activity. In addition to bridging gaps in this healthcare-community transition, virtual programs can support adults living with disabilities and older adults in achieving recommended physical activity guidelines.^31^ The delivery of virtual exercise programs can vary widely in format and structure. Programs may be fully remote, hybrid (combining virtual and in-person sessions), synchronous (i.e., a real-time approach),^32^ asynchronous (i.e., no real-time interaction),^33^^,^^34^ or a combination of both formats.^35^

Previous reviews have assessed the effects, feasibility or safety of virtually delivered exercise to people with stroke,^29^^,^^36^^,^^37^ MS,^28^ PD,^38^ and knee OA.^39^^,^^40^ Despite the growing number of virtual programs, several gaps remain. Most studies have small sample sizes^32^^,^^33^ and focus on single conditions,^32^^,^^33^^,^^41^ limiting generalizability. There is also limited information on how these programs are delivered outside of traditional healthcare settings and the specific strategies used to engage participants safely. Additionally, little is known about the methodological approaches used to evaluate these programs, for example, study designs and outcomes evaluated. To our knowledge, no evidence synthesis has yet been conducted on evaluations of virtual exercise programs targeting mobility limitations delivered outside conventional healthcare or rehabilitation services. Therefore, to address existing gaps and provide guidance for future program design, research, and community implementation, the objectives of this study were to describe the intervention and methodological characteristics in evaluations of virtual exercise programs for adults with mobility limitations.

## 2 Materials and methods

### 2.1 Protocol and registration

We conducted a scoping review following established methodological guidance.^42^^,^^43^ The protocol was registered^44^ and published a priori,^45^ and the PRISMA-ScR^46^ guided this reporting.

### 2.2 Search and information sources

For more information on the methodology, see the published protocol.^45^ Briefly, the search strategy was developed with a librarian using search terms from relevant sources,^47^, ^48^, ^49^ including terms related to adults with mobility limitations, exercise programs, and telehealth. The search strategy is available in appendix A. No date or language limitations were imposed.

We searched Medline, Embase, PEDro, CINAHL, and Scopus in May 2023 and updated the search in January 2025 to identify new studies. We did not search for grey literature due to the potential for incomplete data. After removing duplicates in EndNote™ following Bramer et al.^50^ method for deduplication, study selection was completed using Covidence™.

### 2.3 Study selection

We included peer-reviewed studies meeting the following inclusion criteria: (1) the study includes adults (≥18 years) living in the community, to comprehensively capture literature on mobility limitations across adulthood and reflect conditions affecting mobility throughout the lifespan; (2) the study sample is composed of (at least 75%) people with health conditions associated with mobility limitations (e.g., stroke, OA, PD, MS, frailty); (3) the study evaluated a virtual exercise program, defined as a planned, structured, repetitive physical activity (e.g., aerobic, balance, flexibility, strength, or task-oriented training),^31^ lasting longer than one session, and offering ≥50% live interaction with facilitators or other participants exercising at home; (4) the exercise program targeted some aspect of mobility, defined as tasks or activities involving changing or maintaining body position (i.e., static and dynamic balance), walking, or moving around,^1^ and (5) had a randomized or non-randomized trial, or a before and after single group study design.

We excluded studies meeting the following exclusion criteria: (1) included adults from long-term care or assisted living settings to focus the review on aging in place; (2) exercise was described by authors as therapy, or participants described using clinical terms (i.e., patients) in the article text; (3) limited the intervention to prescribing exercise; (4) published only an abstract, letter, or protocol; and (5) was a systematic or scoping review.

Three trained reviewers (RBdS, JL, and AB) underwent a calibration exercise that involved reviewing the same subset of a random sample of abstracts (n = 50) and full-text (n = 10) articles, with the goal of achieving 80% agreement between reviewers.^46^ Abstracts and full-text were then reviewed in duplicate. The reviewers met regularly to discuss and resolve any discrepancies.^51^

### 2.4 Charting the data

The reviewers piloted data charting on 5% of included articles to optimize accuracy,^51^ after which we incorporated examples from the piloted studies to enhance clarity. Data charting was then conducted in duplicate using Covidence™. We used Google Translate to screen and abstract data from one study written in Korean.

We charted the following information from the included studies: characteristics of the program and program providers; study methodology (e.g., author, year, country, study design, sample size); modes of delivery (e.g., completely remote or hybrid format), terminologies used to characterize the intervention, program duration and frequency, session duration, exercise components, program attendance, effects on balance and mobility, and safety strategies, defined as any instance in which the authors explicitly stated that a given criterion, procedure, or decision was implemented to address participant safety.

### 2.5 Collating, summarizing, and reporting the results

Data were summarized descriptively. To ensure comparability, program attendance is reported as a percentage of total sessions, and when only the number of sessions attended was reported, this value was converted to a percentage. The types of programs were categorized into single or multiple exercise intervention when included two or more types of exercise interventions (e.g., aerobic training, balance training, and strength training). We classified study outcomes using International Classification of Functioning, Disability and Health (ICF)^1^ terminology as (1) body structure and function or impairment; (2) activity or activity limitation (3) participation or participation restriction; (4) personal factor; and (5) environmental factor. Classification was conducted by the first author using the ICF browser^52^ and a previous review,^20^ and verified for appropriateness by the co-authors experienced in using the ICF.

### 2.6 Protocol deviation

During data charting, we found substantial heterogeneity in reporting methods, outcome measures, assessment modes, and time points, preventing calculation of effect sizes as planned.^45^ We therefore reported primary-study findings directly, focusing on pre–post RCT results and outcomes related to balance or mobility per a prior review.^53^

## 3 Results

### 3.1 Selection of sources of evidence

**Fig. 1:**
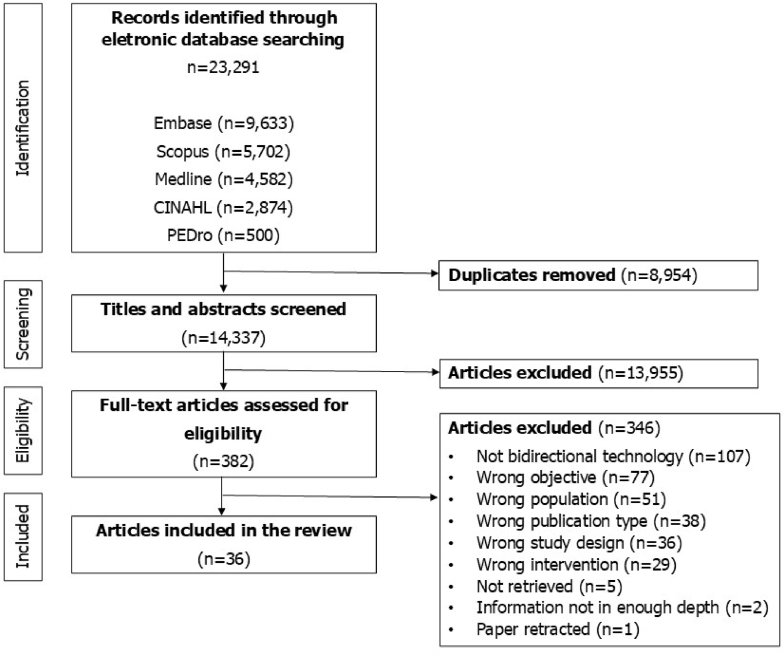
PRISMA flow diagram presenting the study selection process.

Fig. 1 presents the study selection process. After screening 382 full text, we included 36 articles.^54^, ^55^, ^56^, ^57^, ^58^, ^59^, ^60^, ^61^, ^62^, ^63^, ^64^, ^65^, ^66^, ^67^, ^68^, ^69^, ^70^, ^71^, ^72^, ^73^, ^74^, ^75^, ^76^, ^77^, ^78^, ^79^, ^80^, ^81^, ^82^, ^83^, ^84^, ^85^, ^86^, ^87^, ^88^, ^89^.

### 3.2 Characteristics of sources of evidence

Table 1 describes study methodology and exercise program characteristics. All 36 included studies were published after 2017. Thirteen countries were represented: the USA (n = 15, 42%^56^^,^^57^^,^^59^, ^60^, ^61^^,^^68^, ^69^, ^70^^,^^72^^,^^76^^,^^79^^,^^83^^,^^84^^,^^86^^,^^87^), Canada (n = 7, 20%^63,71,73,78,82,85,86 86^), Brazil (n = 3, 8%^54^^,^^58^^,^^88^), Turkey (n = 3, 8%^64^^,^^65^), Korea (n = 2, 6%^62^^,^^89^), Malaysia and Iran (n = 2, 6%^66^^,^^67^), Australia (n = 1, 3%^75^), India (n = 1, 3%^77^), Israel (n = 1, 3%^74^), Portugal (n = 1, 3%^55^), Sweden (n = 1, 3%^81^), and the UK (n = 1, 3%^86^). One study included participants from three countries,^86^ and two included participants from two countries.^66^^,^^67^

**Table 1:** Characteristics of exercise programs.

Program characteristics	Number of studies reporting	No. (%) of studies
Target population	36	
Parkinson's disease		11 (31)
Multiple sclerosis		4 (11)
Stroke		4 (11)
Knee osteoarthritis		3 (8)
Spinal cord injury		3 (8)
Frail older adults		2 (6)
Older adults with mild cognitive impairment		2 (6)
Other^a^		7 (19)
Delivery format	36	
Remote^b^		32 (89)
Hybrid^b^		4 (11)
Platform or software	34	
Zoom		23 (68)
Other^c^		11 (32)
Class duration (minutes)	32	
20-45		5 (16)
60		20 (63)
>60		5 (16)
Other^d^		2 (5)
Classes per week, No.	36	
1		2 (5)
2		17 (47)
3		11 (31)
4		3 (8)
Other^e^		3 (8)
Program duration (weeks)	36	
4		3 (8)
6		7 (19)
8		12 (33)
12		7 (19)
≥16		7 (19)
Type of program	36	
Single exercise intervention		19 (53)
Multiple exercise interventions		17 (47)
Type of exercise intervention^f^	36	
Strength training		16 (44)
Aerobic training		11 (31)
Balance training		7 (19)
Yoga		5 (14)
Mindfulness		4 (11)
Task-oriented training^g^		4 (11)
Flexibility training		3 (8)
Tai Chi		3 (8)
Other^h^		5 (14)
Program format^f^	24	
Group		16 (67)
2-5 people per group		4 (25)
6-10 people per group		5 (31)
>10 people per group		6 (38)
Individual		9 (38)
Program provider per class, No.	29	
1		20 (69)
2		8 (28)
4		1 (3)
Program providers^f^	29	
Registered healthcare professional		19 (66)
Physiotherapist		14 (48)
Other healthcare professional		5 (17)
Fitness professional		11 (38)
Other^i^		3 (10)

a Included individuals with both chronic obstructive pulmonary disease and heart failure, rheumatoid arthritis, traumatic brain injury, individuals using wheelchairs, older adults at risk for Alzheimer's disease and related dementias, and older adults with lower limb amputation.

b Included synchronous (n = 29, 80%), and synchronous and asynchronous (n = 7, 20%).

c Included: Google Meet™, Skype™, YouTube™, WhatsApp™, Team Meetings™.

d Included 10-25 min/class (varied by cohort) and 15-20 min/class.

e First class in-person in 2 studies; first week in-person in 1 study; 2 classes/week during months 1-3, 1 class/week during months 4-6; 2 classes/week during weeks 1-2, 1 class/week during weeks 3-8.

f Categories are not mutually exclusive, some studies included more than one type of exercise intervention, program format, and/or program provider.

g Included tasks such as sitting and standing, reaching, and stepping or walking.

h Included Pilates, Lee Silverman Voice Therapy (LSVT)-BIG, Tai chi, dual-task Tai Ji Quan, console-based exergames (e.g., XBOX 360 Kinect), Wii Fit balance board activities.

i Included LoveYourBrain-trained facilitators, doctoral students, a health educator specialized in adapted physical education, and research staff.

### 3.3 Synthesis of results

#### 3.3.1 Study methodology and target population

The study designs included pre-post study design (n = 14, 39%^54^^,^^55^^,^^57^^,^^61^, ^62^, ^63^, ^64^^,^^71^^,^^78^^,^^79^^,^^82^^,^^84^^,^^86^^,^^88^), randomized controlled trials (RCTs) (n = 10, 28%^56^^,^^59^^,^^60^^,^^65^, ^66^, ^67^^,^^73^^,^^77^^,^^80^^,^^83^), pilot or feasibility randomized trials (n = 7, 19%^68^^,^^70^^,^^72^^,^^76^^,^^81^^,^^85^^,^^87^), non-randomized trials (n = 3, 8%^62^^,^^69^^,^^74^), dose-escalation study design (n = 1, 3%^75^), and longitudinal observation study (n = 1, 3%^58^). Three studies (8%^55^^,^^57^^,^^70^) included a qualitative component.

Authors used a range of terminologies to describe the virtual nature of the programs, most frequently terms were “online” (n = 9, 25%^54^, ^55^, ^56^^,^^63^^,^^66^^,^^68^^,^^76^^,^^79^^,^^83^), “telerehabilitation” (n = 8, 22%^64^^,^^65^^,^^73^^,^^77^^,^^78^^,^^80^^,^^85^^,^^88^), “tele-exercise” (n = 6, 17%^57^^,^^58^^,^^61^^,^^62^^,^^66^^,^^72^), “virtual” (n = 5, 14%^59^^,^^68^^,^^72^^,^^76^^,^^82^), and “remote” (n = 5, 14%^59^^,^^60^^,^^71^^,^^84^^,^^85^).

Sample sizes ranged from 9^71^ to 411^79^ participants, totaling 1825 participants. The median sample size was 27 participants (P_25_ = 20; P_75_ = 41), with most studies (69%, n = 25) including fewer than 35 participants. In all studies, programs included participants from a homogeneous clinical population, most often PD (n = 11, 31%^54^^,^^55^^,^^59^^,^^62^, ^63^, ^64^^,^^70^^,^^77^^,^^80^^,^^86^^,^^88^), MS (n = 4, 11%^65^, ^66^, ^67^, ^68^), stroke (n = 4, 11%^71^^,^^75^^,^^78^^,^^81^), knee OA (n = 3, 8%^84^^,^^85^^,^^87^), spinal cord injury (n = 3, 8%^57^^,^^58^^,^^61^). Across the studies, the mean age of participants ranged from 31^74^ to 76 years,^56^ with participants most commonly aged between 60 and 70 years (n = 15, 42%).

#### 3.3.2 Structure, content and delivery of the exercise programs

Exercise interventions were delivered completely remotely (n = 32, 89%^54^, ^55^, ^56^, ^57^, ^58^, ^59^^,^^61^, ^62^, ^63^, ^64^, ^65^, ^66^, ^67^, ^68^, ^69^^,^^71^^,^^72^^,^^75^^,^^77^, ^78^, ^79^, ^80^, ^81^, ^82^, ^83^, ^84^, ^85^, ^86^, ^87^, ^88^, ^89^) and synchronously (n = 29, 80%^54^, ^55^, ^56^^,^^58^, ^59^, ^60^, ^61^, ^62^, ^63^^,^^65^, ^66^, ^67^, ^68^, ^69^, ^70^, ^71^, ^72^^,^^74^, ^75^, ^76^^,^^78^^,^^80^^,^^83^, ^84^, ^85^, ^86^, ^87^, ^88^, ^89^), through Zoom™ (n = 23/34, 68%^54^, ^55^, ^56^^,^^59^^,^^61^, ^62^, ^63^^,^^65^, ^66^, ^67^, ^68^^,^^71^^,^^72^^,^^74^^,^^76^^,^^79^^,^^80^^,^^83^, ^84^, ^85^, ^86^, ^87^^,^^89^). Most programs (n = 20/32, 63%)^54^, ^55^, ^56^^,^^59^^,^^61^^,^^63^, ^64^, ^65^, ^66^, ^67^, ^68^, ^69^^,^^71^^,^^74^^,^^76^^,^^77^^,^^80^^,^^84^^,^^88^^,^^89^ had a class duration of 60 min, while five (16%)^57^^,^^62^^,^^81^^,^^83^^,^^86^ had shorter sessions (20–45 min) and five (16%)^72^^,^^73^^,^^78^^,^^79^^,^^87^ had longer sessions (>60 min). The majority of programs offered two classes per week (n = 17, 47%),^54^, ^55^, ^56^, ^57^^,^^59^^,^^61^^,^^62^^,^^68^^,^^69^^,^^71^^,^^74^^,^^76^^,^^78^^,^^79^^,^^83^^,^^86^^,^^89^ followed by three classes per week (n = 11, 31%).^58^^,^^63^^,^^65^, ^66^, ^67^^,^^70^^,^^72^^,^^73^^,^^75^^,^^84^^,^^88^ Program duration also differed, with twelve (33%) studies implementing an 8-week program,^54^^,^^57^^,^^59^^,^^61^^,^^66^^,^^67^^,^^69^^,^^70^^,^^73^^,^^75^^,^^82^^,^^87^ while seven studies (19%) each implemented programs lasting 6,^65^^,^^72^^,^^77^^,^^79^^,^^85^^,^^86^^,^^89^ 12,^62,63,68,71,74,83,84^ or 16 weeks or more.^55^^,^^56^^,^^58^^,^^60^^,^^76^^,^^81^^,^^88^ Programs were single (n = 19, 53%) or multiple exercise intervention (n = 17, 47%). Strength training was the most frequently included modality (n = 16, 44%),^57^, ^58^, ^59^, ^60^, ^61^, ^62^^,^^68^^,^^70^^,^^71^^,^^74^^,^^75^^,^^77^^,^^81^^,^^84^^,^^87^^,^^89^ followed by aerobic training (n = 11, 31%)^57^^,^^59^, ^60^, ^61^^,^^70^, ^71^, ^72^^,^^77^^,^^81^^,^^84^^,^^89^ and balance training (n = 7, 19%).^59^^,^^71^, ^72^, ^73^^,^^77^^,^^82^^,^^84^ Among the 19 studies that reported attendance, mean attendance was 82% (range: 45.1%^58^ - 100%^85^), with 12 studies^54^^,^^59^^,^^65^^,^^66^^,^^69^^,^^70^^,^^73^^,^^75^^,^^84^, ^85^, ^86^^,^^88^ reporting attendance above 85%.

Of the 24 studies that reported program format, programs were delivered in groups (n = 16/24, 67%)^54^^,^^57^^,^^59^^,^^61^^,^^63^^,^^68^^,^^69^^,^^71^^,^^73^^,^^76^^,^^78^^,^^79^^,^^81^^,^^83^^,^^87^^,^^88^ and/or individual-based approaches (n = 9/24, 38%).^64^^,^^65^^,^^70^^,^^72^^,^^80^, ^81^, ^82^^,^^85^^,^^86^ Group sizes varied, with 31% (5/16) including groups of 6–10, and 38% (6/16) including larger groups of more than 10 participants. Most programs were led by a single provider (n = 20/29, 69%). Regarding the professional background of program providers delivering the interventions, 57% of studies reported the involvement of registered healthcare professionals, of which 39% were physiotherapists and 14% were other healthcare professional including a kinesiologist,^71^^,^^85^ exercise physiologist,^60^ registered nurse,^62^ and sports rehabilitation expert.^62^ Fitness professionals delivered the intervention in 31% of studies, including a certified yoga instructor,^66^^,^^67^^,^^69^^,^^83^ Tai Chi instructor,^63^ and a dance instructor who was also a physiotherapist.^54^

#### 3.3.3 Strategies used to optimize safety

Thirteen studies (36%) reported safety strategies. These included providing safety manuals with setup guidance and troubleshooting^68^; promoting proper form within individual limits^74^; screening exercise readiness with a standardized tool and requiring medical clearance^61^; conducting pre-session safety checks^57^; educating participants on safe space setup and requiring physician referral for high-intensity exercise^59^; mandating camera use, monitoring adverse events, distributing fall-prevention materials, and involving caregivers or medical personnel^62^; inspecting home environments via Zoom and advising on lighting^72^; confirming equipment and space safety before sessions^83^; requiring caregiver supervision during assessments and co-developing safe plans^78^; sending safety video tutorials and providing technical support^54^; advising hand supports to reduce fall risk^73^; providing real-time feedback during videoconferencing^88^; and conducting video safety screenings and supervised-session checks.^81^

#### 3.3.4 Outcomes evaluated in each study

Table 2 provides a classification of study outcomes for participants. Among 26 (72%) studies reporting outcome assessment methods, 11 studies (42%)^59^^,^^63^, ^64^, ^65^, ^66^^,^^68^^,^^69^^,^^74^^,^^75^^,^^80^^,^^83^ conducted assessments in person, 11 (42%)^54^^,^^56^^,^^67^^,^^69^^,^^72^^,^^76^^,^^78^^,^^79^^,^^82^^,^^85^^,^^87^ used remote methods, and 4 studies (15%)^60^^,^^71^^,^^84^^,^^88^ used a hybrid approach combining both in-person and remote assessments. For program participants, studies most commonly evaluated outcomes classified as body structure and function, followed by activity and participation. Among the outcomes categorized under the body structure and function, muscle strength was the most frequently measured outcome within this domain, reported in 7 studies (19%). Depression was assessed in 6 studies (17%), while cognition and fatigue were each reported as outcomes in 5 studies (14%). In the activity domain, physical function was evaluated in 10 studies (28%), while balance (functional) was evaluated in 8 studies (22%). In addition, studies assessed non-ICF outcomes, most notably health-related quality of life (HRQL), which was reported in 16 studies (44%).

**Table 2:** Classification of study outcomes using the International Classification of Functioning, Disability and Health (ICF).

ICF category (n, % of studies in the category)	Study outcome	No. (%) of studies with outcome
Body structure and function (n = 24, 67%)	Muscle strength	7 (19)
	Depression	6 (17)
	Cognition	5 (14)
	Fatigue	5 (14)
	Balance confidence	3 (8)
	Cardiovascular fitness	3 (8)
	Pain	3 (8)
	Sleep	3 (8)
	Blood pressure	2 (6)
	Body composition	2 (6)
	Range of motion	2 (6)
	Anxiety	1 (3)
	Resilience	1 (3)
Activity (n = 21, 58%)	Physical function	10 (28)
	Balance (functional)	8 (22)
	Mobility	4 (11)
	Walking distance/endurance	3 (8)
	Activities of daily living	1 (3)
	Walking speed	1 (3)
Participation (n = 6, 17%)	Physical activity	6 (17)
Contextual factors: personal (n = 2, 5%)	Metabolic syndrome	1 (3)
	Height	1 (3)
	Weight	1 (3)
Non-ICF outcomes (n = 17, 47%)	Health-related quality of life	16 (44)
	Well-being	2 (6)
	Falls	1 (3)

#### 3.3.5 Overview of intervention effects on balance and mobility

Supplementary table (appendix A) summarizes the pre- and post-intervention results for balance and mobility outcome measures reported in seven RCTs.^56^^,^^59^^,^^65^^,^^67^^,^^73^^,^^80^^,^^83^ Outcomes were assessed from baseline up to time points ranging between 4^73,80^ and 24 weeks.^56^ Common balance tests included the Mini-BESTest,^59^^,^^80^ the Berg balance scale,^65^ the four-step square test,^73^ while mobility measures included the Timed up and go^56^^,^^80^^,^^83^ 6-min walk test,^59^^,^^65^ 2-min walk test.^73^ Most RCTs reported improvements in intervention groups versus controls, though effect sizes and consistency varied. Heterogeneity in interventions, duration, and measures limits direct comparability.

Of the seven RCTs, five included fewer than 20 participants per intervention group, with the exception of two trials with larger sample sizes. Tao^73^ conducted a two-group RCT in 71 older adults with lower-limb amputation, comparing Wii Fit balance-board activities with an attention control using Big Brain Academy Wii-based cognitive activities. Outcomes were assessed at 9 weeks, which showed effect size of 7.47% in balance confidence assessed with the Activities-specific Balance Confidence. Li^56^ conducted a three-group RCT in 318 older adults with mild cognitive impairment, comparing Cognitively Enhanced Tai Ji Quan, Standard Tai Ji Quan, and stretching exercise. Outcomes were assessed at 24 weeks, and both Tai Ji interventions showed statistically significant improvements compared with stretching in Timed Up and Go time (−1.9 and −2.2 s for cognitively enhanced and standard Tai Ji, respectively), 30-s chair stands (1.7 and 2.1 repetitions), and 4-Stage Balance Test scores (.8 and 1.0 points), while differences between the two Tai Ji groups were not statistically significant.

## 4 Discussion

This scoping review provides an overview of 36 studies evaluating virtual exercise programs for adults with mobility limitations. Most studies involved participants with PD, MS, or stroke. Programs were commonly delivered live via Zoom™, with 1-h group classes held twice weekly for 8 weeks, typically led by physiotherapists and focused on strength and aerobic training. Safety strategies varied but included pre-screening, home setup guidance, caregiver support, and real-time supervision. HRQL and physical function were the most commonly assessed outcomes. RCTs generally showed positive effects on balance and mobility, though intervention and outcome variability limited comparability.

### 4.1 Target population and outcomes evaluated

In our review, studies had a median sample size of 27 participants, which is smaller than those reported in other reviews of virtual exercise programs for people with stroke,^29^ people with multiple sclerosis,^28^ or older adults (median sample size: 38 - 50), and reviews of in-person programs targeting people with neurological conditions (median sample size: 45), and older adults (median sample size: 148). This likely reflects the predominance of early-phase study designs in the current literature, as many included studies were pre–post or pilot/feasibility trials, which are typically conducted with smaller samples to assess safety, acceptability, and implementation feasibility before larger-scale trials are undertaken.^90^ Thus, relatively small sample sizes may reflect that the field of experimental research evaluating virtual exercise programs is developing rather than the scalability potential of virtual delivery itself. Notably, all 36 included studies were published after 2017, reflecting the relatively recent growth of research on virtual exercise delivery over the last decade.

Our review highlighted that evaluations of virtual exercise programs more commonly enrol people with a specific health condition rather than people at a specific functional level regardless of the underlying cause. This population specific approach aligns with findings from other reviews of virtual exercise interventions for people with disabilities^91^ and of in-person community exercise programs delivered by fitness instructors for people with neurological conditions.^20^ Condition-specific programs may enable tailored interventions and foster a sense of shared identity and community among participants, as described by people post-stroke.^92^ Important, this preference was identified in the context of in-person programs, and it remains unclear to what extent similar dynamics apply to virtual exercise settings. In addition, reliance on condition-specific programming may pose challenges in settings with smaller populations, such as rural areas, where there may not be enough participants to form groups based on a single health condition.

In terms of outcomes evaluated, the broad range of outcomes across studies suggests that virtual exercise interventions are increasingly conceptualized as multidimensional programs offering benefits across physical, psychological, and social domains. Beyond direct physical benefits from exercise components (e.g., strength, balance, and aerobic training), the virtual format may also provide psychosocial benefits by enabling participation for individuals who face barriers to attending in-person programs, such as transportation limitations, distance, or adverse weather conditions, and by providing opportunities for social interaction that may mitigate isolation. We identified that health-related quality of life (HRQL) was the most commonly assessed outcome across studies, alongside physical outcomes such as physical function, balance, and strength, as well as psychosocial outcomes including depression. One possible reason for the prominence of HRQL is that it is typically measured using questionnaire-based scales,^93^ which are more feasible to administer in virtual settings than performance-based assessments of mobility or balance. Despite the prominence of HRQL in this review and in other reviews of virtual exercise programs,^91^ the variability of measurement tools, including both generic and condition-specific scales (e.g., Leeds multiple sclerosis quality of life scale, quality of life scale after brain injury, Parkinson's disease quality of life questionnaire-39), may pose challenges for conducting systematic reviews and meta-analyses on effectiveness of virtual exercise programs on HRQL.

### 4.2 Structure, content, and delivery of the exercise programs

We found that the most common (33%) program duration was 8 weeks, with only 19% of programs lasting 12 weeks, consistent with a review of virtual group-based programs for older adults.^94^ In contrast, a review of in-person group-based programs for people with neurological conditions found that most programs lasted 12 weeks.^20^ This shift toward shorter program durations in virtual interventions may represent an emerging trend in virtual exercise delivery and may reflect preferences for shorter, flexible formats that enhance feasibility and scalability. The core types of exercise in our review, such as aerobic, balance, and strength training, were similar to those observed in other reviews of virtual^94^ and in-person programs.^20^ Notably, compared to these reviews,^20^^,^^94^ studies in our review also included a broader range of modalities, including yoga, Tai Chi, and Pilates. This may be related to the minimal space and equipment requirements of these approaches, making them particularly well suited to home-based virtual delivery.

In the majority of included studies (66%), healthcare professionals were responsible for delivering the program. Similarly, registered or highly experienced exercise professionals, including physical therapists, led over half of virtual programs in a previous review.^94^ This high involvement of healthcare professionals in virtual delivery may reflect the specific challenges of remote supervision: without the ability to provide hands-on assistance, instructors rely on verbal cueing and visual monitoring, which may increase perceived liability and reinforce the need for clinical oversight. Indeed, regarding safety strategies, studies primarily focused on two key aspects: (1) participant preparation and home environment setup, including participant education through manuals or tutorials and pre-session safety checks to ensure adequate space, appropriate clothing and footwear, and access to hand support; and (2) exercise execution, addressed through real-time monitoring via videoconferencing by program providers, encouragement of self-pacing, or the presence of an on-site caregiver. These findings suggest a shift from greater reliance on in-person components, such as initial exercise prescriptions and onsite non-healthcare supervision, reported in a previous review.^36^

Notably, only half of the included studies reported program attendance. Among these, mean attendance was 82%, with 12 studies reporting attendance above 85%. These findings are consistent with previous work reporting a mean attendance of 82% in virtual group-based programs for older adults.^94^ By comparison, in-person community programs have reported a lower mean attendance of 69%,^20^ suggesting that virtual programs can achieve higher attendance rates than those of traditional in-person programs. This difference may partly reflect barriers associated with attending in-person sessions, such as transportation needs or disruptions due to inclement weather,^21^^,^^22^^,^^24^ barriers that can be mitigated through virtual delivery models.

### 4.3 Strengths and limitations

This scoping review is the first to comprehensively map the characteristics, delivery modes, and safety strategies of virtual community exercise programs for adults with mobility limitations. Some key strengths of our review are in the use of established scoping review methodology, a comprehensive search strategy developed with a research librarian, and application of a rigorous selection and data extraction process conducted by trained reviewers. Additionally, by classifying outcomes according to the ICF, our review offers a structured understanding of the benefits and targets of virtual exercise programs. Finally, by reporting strategies used to optimize safety, an area often underreported in previous reviews, our findings provide practical guidance for researchers, clinicians, and program providers aiming to scale virtual interventions in a safe and inclusive manner.

Some limitations must be acknowledged. First, the search strategy focused on conditions commonly associated with mobility and balance limitations (e.g., stroke, PD, OA), may have excluded studies involving otherwise healthy older adults with similar functional limitations. This decision was based on the challenge of consistently identifying participants with mobility limitations across studies, given variation in outcome measures and cut-off scores, which would have compromised the feasibility and clarity of our screening process. Second, given the evolving nature of virtual exercise delivery, especially in the context of the COVID-19 pandemic, our findings may reflect programs designed in response to short-term constraints rather than long-term implementation strategies. Lastly, authors of included studies used a wide range of terms to describe the virtual nature and focus of the exercise programs, with some using the term “telerehabilitation” (i.e., use of technology to provide remote rehabilitation activities^95^), which makes it challenging to determine whether programs are community-based or part of formal rehabilitation services. Furthermore, the inconsistent use of terminology poses challenges for evidence synthesis efforts, as it may lead to the omission of relevant studies during database searches or misclassification of program types. Standardizing terminology in future research would improve clarity and enhance the ability to systematically identify, compare, and build upon existing literature.^96^

### 4.4 Implications for future research

While the identified predominance of homogeneous, condition-specific samples enables tailored programming and careful risk management, it may limit generalizability to community settings that typically serve individuals with diverse diagnoses and varying levels of mobility limitation. Future research should evaluate more heterogeneous cohorts that better reflect real-world practice. At the same time, the breadth of outcomes assessed, including balance, mobility, strength, HRQL, depression, and functional performance, suggests that virtual exercise interventions may yield multidimensional benefits for various populations. However, current variability in outcome measures constrains synthesis. Adoption of core outcome sets and greater harmonization of measurement tools will be essential to support comparison across studies and potential meta-analysis, particularly in key outcomes for this population such as balance and mobility.

In addition, much of the literature remains based in feasibility and pilot trials with modest sample sizes. Our findings show that virtual exercise delivery appears feasible, safe, beneficial, and with high attendance rates (often exceeding 85%). These findings suggest that the field is well positioned to move toward larger-scale effectiveness studies to establish definitive health benefits, and hybrid effectiveness–implementation designs to further support scalability and integration into community-based health systems.

Lastly, given the complexity of virtual exercise interventions, future research should also incorporate robust process evaluations to examine implementation fidelity, mechanisms of impact, and contextual influences.^97^ Qualitative methodologies are particularly valuable for understanding participants lived experiences, identifying barriers and facilitators to participation,^98^ and exploring how delivery models can promote exercise participation across diverse settings. These approaches are essential to advancing the accessibility, scalability, and sustainability of virtual exercise programs for adults with mobility limitations.

## 5 Conclusion

This scoping review provides a comprehensive synthesis of virtual community exercise programs for adults with mobility limitations. Most programs were delivered synchronously via Zoom™ by physiotherapists, with 60-min group sessions offered twice weekly over 8 weeks, focusing on strength, aerobic, and emerging mind–body modalities. Safety strategies included pre-screening, home setup guidance, and real-time supervision. Health-related quality of life and physical function were the most commonly assessed outcomes. The literature predominantly enrolled homogeneous, health condition-specific groups, and many studies were early-phase feasibility or pilot trials with small sample sizes. Nevertheless, our findings indicate that virtual programs appear feasible and safe, achieve high attendance rates, and may be beneficial across physical, psychological, and social domains. These findings support the progression to larger, well-powered trials, adoption of standardized outcomes, and inclusion of heterogeneous populations to evaluate scalability, sustained engagement, and real-world effectiveness.

## Author contributions

**Renato Barbosa dos Santos:** Conceptualization, Methodology, Project Administration, Data Curation, Formal Analysis, Investigation, Writing – original draft, Writing – review & editing; **Jing Lin, Anchal Badwal:** Data Curation, Writing – review & editing; **Hardeep Singh, Susan Jaglal, Chavon Niles:** Methodology, Writing – review & editing; **Nancy Salbach:** Conceptualization, Methodology, Supervision, Project Administration, Writing – review & editing.

## Declaration of funding

Renato Barbosa dos Santos received the Physiotherapy Foundation of Canada Ann Collins Whitmore Memorial Scholarship, and is a fellow in the EPIC-AT Health Research Training Platform. Nancy Salbach holds the Toronto Rehabilitation Institute Chair at the University of Toronto. The funders had no role in the design, execution, analysis, interpretation of data, or writing of this study.

## Declartion of competing interest

The authors report no conflicts of interest.

## References

[1] International Classification of Functioning Disability and health (ICF). https://www.who.int/standards/classifications/international-classification-of-functioning-disability-and-health.

[2] Global report on health equity for persons with disabilities. https://www.who.int/teams/noncommunicable-diseases/sensory-functions-disability-and-rehabilitation/global-report-on-health-equity-for-persons-with-disabilities.

[3] Louie D.R. (2022). Prevalence of walking limitation after acute stroke and its impact on discharge to home. Phys Ther.

[4] King L.K., Kendzerska T., Waugh E.J., Hawker G.A. (2018). Impact of osteoarthritis on difficulty walking: a population-based study. Arthritis Care Res.

[5] Martin C.L. (2006). Gait and balance impairment in early multiple sclerosis in the absence of clinical disability. Mult Scler J.

[6] Lindh-Rengifo M., Jonasson S.B., Ullén S., Mattsson-Carlgren N., Nilsson M.H. (2021). Perceived walking difficulties in Parkinson's disease – predictors and changes over time. BMC Geriatr.

[7] Tulsky D.S. (2022). Physical function recovery trajectories after spinal cord injury. Arch Phys Med Rehabil.

[8] Tolea M.I., Morris J.C., Galvin J.E. (2016). Trajectory of mobility decline by type of dementia. Alzheimer Dis Assoc Disord.

[9] Davis D.H.J., Rockwood M.R.H., Mitnitski A.B., Rockwood K. (2011). Impairments in mobility and balance in relation to frailty. Arch Gerontol Geriatr.

[10] Chisari C., Venturi M., Bertolucci F., Fanciullacci C., Rossi B. (2014). Benefits of an intensive task-oriented circuit training in multiple sclerosis patients with mild disability. NeuroRehabilitation.

[11] Wevers L., van de Port I., Vermue M., Mead G., Kwakkel G. (2009). Effects of task-oriented circuit class training on walking competency after stroke. Stroke.

[12] English C., Hillier S.L., Lynch E.A. (2017). Circuit class therapy for improving mobility after stroke. Cochrane Database Syst Rev.

[13] Skou S.T., Roos E.M. (2017). Good life with osteoArthritis in Denmark (GLA:D^TM^): evidence-based education and supervised neuromuscular exercise delivered by certified physiotherapists nationwide. BMC Muscoskelet Disord.

[14] Aravind G. (2022). Community-based exercise programs incorporating healthcare-community partnerships to improve function post-stroke: feasibility of a 2-group randomized controlled trial. Pilot Feasibility Stud.

[15] Salbach N.M., Howe J.-A., Brunton K., Salisbury K., Bodiam L. (2014). Partnering to increase access to community exercise programs for people with stroke, acquired brain injury, and multiple sclerosis. J Phys Activ Health.

[16] Harrington R. (2010). A community-based exercise and education scheme for stroke survivors: a randomized controlled trial and economic evaluation. Clin Rehabil.

[17] Stuart M. (2019). Adaptive physical activity for stroke: an early-stage randomized controlled trial in the United States. Neurorehabilitation Neural Repair.

[18] Dean S.G. (2018). Community-based rehabilitation training after stroke: results of a pilot randomised controlled trial (ReTrain) investigating acceptability and feasibility. BMJ Open.

[19] Pang M.Y.C., Eng J.J., Dawson A.S., McKay H.A., Harris J.E. (2005). A community-based fitness and mobility exercise (FAME) program for older adults with chronic stroke: a randomized controlled trial. J Am Geriatr Soc.

[20] Merali S., Cameron J.I., Barclay R., Salbach N.M. (2016). Characterising community exercise programmes delivered by fitness instructors for people with neurological conditions: a scoping review. Health Soc Care Community.

[21] Salbach N.M., Howe J.-A., Baldry D., Merali S., Munce S.E.P. (2018). Considerations for expanding community exercise programs incorporating a healthcare-recreation partnership for people with balance and mobility limitations: a mixed methods evaluation. BMC Res Notes.

[22] Prakash P., Scott T.F., Baser S.M., Leichliter T., Schramke C.J. (2021). Self-reported barriers to exercise and factors impacting participation in exercise in patients with parkinson's disease. Mov Disord Clin Pract.

[23] Dobson F. (2016). Barriers and facilitators to exercise participation in people with hip And/or knee osteoarthritis: synthesis of the literature using behavior change theory. Am J Phys Med Rehabil.

[24] Kokorelias K.M. (2022). Moving through COVID-19: perspectives of older adults in the getting older adults outdoors study. J Aging Phys Activ.

[25] Merali S., Cameron J.I., Barclay R., Salbach N.M. (2020). Experiences of people with stroke and multiple sclerosis and caregivers of a community exercise programme involving a healthcare-recreation partnership. Disabil Rehabil.

[26] Barbosa Dos Santos R. (2025). Feasibility, safety, and potential benefit of a virtual, community-based, task-oriented exercise program (TIME^TM^ at home) for people with balance and mobility limitations: a pre-post feasibility study. Disabil Rehabil.

[27] Salbach N.M. (2022). Canadian stroke best practice recommendations: virtual stroke rehabilitation interim consensus statement 2022. Am J Phys Med Rehabil.

[28] Khan F., Amatya B., Kesselring J., Galea M. (2015). Telerehabilitation for persons with multiple sclerosis. Cochrane Database Syst Rev.

[29] Laver K.E. (2020). Telerehabilitation services for stroke. Cochrane Database Syst Rev.

[30] Gavin J.P. (2025). Maintaining physical activity in people with long-term conditions following engagement in physical activity referral schemes: barriers, enablers, and intervention strategies. Int J Behav Nutr Phys Activ.

[31] (2020). WHO Guidelines on Physical Activity and Sedentary Behaviour.

[32] Gell N.M. (2022). Remotely delivered exercise to older rural cancer survivors: a randomized controlled pilot trial. J Cancer Surviv Res Pract.

[33] Torriani-Pasin C. (2021). Adherence rate, barriers to attend, safety, and overall experience of a remote physical exercise program during the COVID-19 pandemic for individuals after stroke. Front Psychol.

[34] Kannan M. (2019). Evaluation of a web-based fall prevention program among people with multiple sclerosis. Mult Scler Relat Disord.

[35] Galloway M. (2019). The feasibility of a telehealth exercise program aimed at increasing cardiorespiratory fitness for people after stroke. Int J Telerehabilitation.

[36] Ramage E.R. (2021). Look before you leap: interventions supervised via telehealth involving activities in weight-bearing or standing positions for people after stroke—A scoping review. Phys Ther.

[37] Knepley K.D. (2021). Impact of telerehabilitation for stroke-related deficits. Telemed E-Health.

[38] Vellata C. (2021). Effectiveness of telerehabilitation on motor impairments, non-motor symptoms and compliance in patients with parkinson's disease: a systematic review. Front Neurol.

[39] Schäfer A.G.M., Zalpour C., von Piekartz H., Hall T.M., Paelke V. (2018). The efficacy of electronic health-supported home exercise interventions for patients with osteoarthritis of the knee: systematic review. J Med Internet Res.

[40] Chen T., Or C.K., Chen J. (2021). Effects of technology-supported exercise programs on the knee pain, physical function, and quality of life of individuals with knee osteoarthritis and/or chronic knee pain: a systematic review and meta-analysis of randomized controlled trials. J Am Med Inform Assoc JAMIA.

[41] Torriani‐Pasin C. (2022). Adherence rate, barriers to attend, safety and overall experience of a physical exercise program via telemonitoring during COVID‐19 pandemic for indivJ Am Med Inform Assoc JAMIAiduals with Parkinson's disease: a feasibility study. Physiother Res Int.

[42] Levac D., Colquhoun H., O'Brien K.K. (2010). Scoping studies: advancing the methodology. Implement Sci.

[43] Arksey H., O'Malley L. (2005). Scoping studies: towards a methodological framework. Int J Soc Res Methodol.

[44] Santos R. B. dos (2023). Evaluations of virtual exercise programs for adults with mobility limitations: a scoping review protocol incorporating an equity-lens to inform the development of strategies to optimize participation of underrepresented groups.

[45] Santos R. B. dos (2024). Evaluations of virtual exercise programmes for adults with mobility limitations: a scoping review protocol incorporating an equity lens to inform the development of strategies to optimise participation of under-represented groups. BMJ Open.

[46] Tricco A.C. (2018). PRISMA extension for scoping reviews (PRISMA-ScR): checklist and explanation. Ann Intern Med.

[47] French B. (2016). Repetitive task training for improving functional ability after stroke. Cochrane Database Syst Rev.

[48] Saunders D.H. (2020). Physical fitness training for stroke patients. Cochrane Database Syst Rev.

[49] CADTH COVID-19 Search Strings CADTH Covid-19 evidence portal. https://covid.cadth.ca/literature-searching-tools/cadth-covid-19-search-strings/.

[50] Bramer W.M., Giustini D., de Jonge G.B., Holland L., Bekhuis T. (2016). De-duplication of database search results for systematic reviews in EndNote. J Med Libr Assoc JMLA.

[51] Pollock, D. et al. Recommendations for the extraction, analysis, and presentation of results in scoping reviews. JBI Evid Synth 10.11124/JBIES doi:10.11124/JBIES-22-00123. 10.11124/JBIES-22-00123 36081365

[52] International Classification of Functioning Disability and health. https://icd.who.int/browse/2025-01/icf/en.

[53] dos Santos R.B. (2023). Standardized tools for assessing balance and mobility in stroke clinical practice guidelines worldwide: a scoping review. Front Rehabil Sci.

[54] Pinto C. (2023). A safe and feasible online dance intervention for older adults with and without parkinson's disease. J Dance Med Sci.

[55] Domingos J., Dean J., Fernandes J.B., Godinho C. (2022). An online dual-task cognitive and motor exercise program for individuals with parkinson disease (PD3 move program): acceptability study. JMIR Aging.

[56] Li F., Harmer P., Eckstrom E., Fitzgerald K., Winters-Stone K. (2023). Clinical effectiveness of cognitively enhanced Tai Ji quan training on global cognition and dual-task performance during walking in older adults with mild cognitive impairment or self-reported memory concerns: a randomized controlled trial. Ann Intern Med.

[57] Baehr L.A., Kaimal G., Bruneau M., Finley M. (2023). Development and feasibility of a group tele-exercise program for individuals with spinal cord injury. J Neurol Phys Ther.

[58] Gomes Costa R.R., Dorneles J.R., Veloso J.H.C.L., Gonçalves C.W.P., Ribeiro Neto F. (2025). Does tele-exercise training for tetraplegia meet the spinal cord injury-specific physical activity guidelines? A 7-month longitudinal study. J Telemed Telecare.

[59] Fishel S.C., Hotchkiss M.E., McNamara C.A., Sevilla K.M., Brown S.A. (2024). Effect of group virtual exercise on people with Parkinson's disease: a randomized controlled trial. Physiother Theory Pract.

[60] Andonian B.J. (2024). Effect of remotely supervised weight loss and exercise training versus lifestyle counseling on cardiovascular risk and clinical outcomes in older adults with rheumatoid arthritis: a randomized controlled trial. ACR Open Rheumatol.

[61] Baehr L.A. (2024). Effect of tele-exercise to promote empowered movement for individuals with spinal cord injury (TEEMS) program on physical activity determinants and behavior: a mixed methods assessment. Arch Phys Med Rehabil.

[62] Ha J. (2024). Effectiveness of live-streaming tele-exercise intervention in patients with parkinson's disease: a pilot study. J Mov Disord.

[63] Law N.-Y., Li J.X. (2023). Effects of a 12-week online Tai chi intervention on gait and postural stability in individuals with Parkinson's disease. Sports Med Health Sci.

[64] Ekmekyapar Fırat Y., Turgay T., Soğan S.S., Günel Karadeniz P. (2023). Effects of LSVT-BIG via telerehabilitation on non-motor and motor symptoms and quality of life in Parkinson's disease. Acta Neurol Belg.

[65] Eldemir K. (2024). Effects of Pilates-based telerehabilitation on physical performance and quality of life in patients with multiple sclerosis. Disabil Rehabil.

[66] Najafi P. (2023). Effects of tele-exercise training on physical and mental health and quality of life in multiple sclerosis: do the effects differ by modality and clinical disease course?. Mult Scler Relat Disord.

[67] Najafi P. (2023). Effects of tele-pilates and Tele-Yoga on biochemicals, physical, and psychological parameters of females with multiple sclerosis. J Clin Med.

[68] Adamson B. (2025). Evaluating the impact of seated pilates on functional outcomes among those with mild, moderate, and severe multiple sclerosis impairment: a pilot feasibility trial. Adapt Phys Act Q (APAQ).

[69] Donesky D., Selman L., McDermott K., Citron T., Howie-Esquivel J. (2017). Evaluation of the feasibility of a home-based TeleYoga intervention in participants with both chronic obstructive pulmonary disease and heart failure. J Alternative Compl Med.

[70] Lai B. (2020). Exploring the uptake and implementation of tele-monitored home-exercise programmes in adults with Parkinson's disease: a mixed-methods pilot study. J Telemed Telecare.

[71] Gagnon M.-A., Batcho C.S., Bird M.-L., Labbé B., Best K.L. (2023). Feasibility of a remotely supervised home-based group eHealth fitness and mobility exercise program for stroke: French-Canadian version preliminary study. Top Stroke Rehabil.

[72] Kannan L. (2024). Gaming-based tele-exercise program to improve physical function in frail older adults: feasibility randomized controlled trial. J Med Internet Res.

[73] Tao G. (2022). Group-based telerehabilitation intervention using wii fit to improve walking in older adults with lower limb amputation (WiiNWalk): a randomized control trial. Clin Rehabil.

[74] Aviram R., Parmet Y., Bar-Haim S. (2024). Health-related effects of real-time circuit tele-training and gym resistance-aerobic training in ambulatory adults with cerebral Palsy.

[75] Galloway M. (2023). How little is enough? The feasibility of conducting a dose-escalation study for exercise training in people with stroke. J Stroke Cerebrovasc Dis.

[76] Li F., Harmer P., Voit J., Chou L.-S. (2021). Implementing an online virtual falls prevention intervention during a public health pandemic for older adults with mild cognitive impairment: a feasibility trial. Clin Interv Aging.

[77] Deepa S. (2023). Improving work life balance among female educationists during the COVID-19 lockdown. Work.

[78] Park S. (2024). Investigating the telerehabilitation with aims to improve lower extremity recovery poststroke program: a feasibility study. Phys Ther.

[79] Callahan C.E., Beisecker L., Zeller S., Donnelly K.Z. (2023). LoveYourBrain mindset: feasibility, acceptability, usability, and effectiveness of an online yoga, mindfulness, and psychoeducation intervention for people with traumatic brain injury. Brain Inj.

[80] Kaya Aytutuldu G., Ersoz Huseyinsinoglu B., Karagoz Sakalli N., Sen A., Yeldan I. (2024). LSVT® BIG versus progressive structured mobility training through synchronous telerehabilitation in Parkinson's disease: a randomized controlled trial. Neurol Sci.

[81] Thurston C. (2025). Mobile health delivered physical activity after mild stroke or transient ischemic attack: is it feasible and acceptable?. Int J Stroke.

[82] Wang E. (2022). MoveStrong at home: a feasibility study of a model for remote delivery of functional strength and balance training combined with nutrition education for older pre-frail and frail adults. Appl Physiol Nutr Metab.

[83] Park J., Wiese L.A.K., Holt J. (2023). Online chair yoga and digital learning for rural underserved older adults at risk for alzheimer's disease and related dementias. Clin Gerontol.

[84] Patel K.V., Hoffman E.V., Phelan E.A., Gell N.M. (2022). Remotely delivered exercise to rural older adults with knee osteoarthritis: a pilot study. ACR Open Rheumatol.

[85] Charlton J.M., Krowchuk N.M., Eng J.J., Li L.C., Hunt M.A. (2023). Remotely delivered, individualized, and self-directed gait modification for knee osteoarthritis: a pilot trial. Clin Biomech.

[86] James-Palmer A.M., Daneault J.-F. (2022). Tele-yoga for the management of Parkinson disease: a safety and feasibility trial. Digit Health.

[87] Shah N. (2024). Telehealth mindful exercise for people with knee osteoarthritis: a decentralized feasibility randomized controlled trial. Osteoarthr Carti Open.

[88] Tardelli E. (2023). Telerehabilitation during social distancing for people with Parkinson's disease: a retrospective study. Acta Neurol Belg.

[89] Han K.-M., Lee S.-Y., Kim J.Y. (2022). The effect of a 6-week non-contact exercise program on body composition and physical fitness in persons with physical disabilities using wheelchairs. Exerc Sci.

[90] Eldridge S.M. (2016). Defining feasibility and pilot studies in preparation for randomised controlled trials: development of a conceptual framework. PLoS One.

[91] Mintz M. (2024). Current trends in virtual exercise interventions among people with disabilities: a scoping review. Arch Rehabil Res Clin Transl.

[92] Bird M.-L. (2019). Building a bridge to the community: an integrated knowledge translation approach to improving participation in community-based exercise for people after stroke. Phys Ther.

[93] Andresen E.M., Meyers A.R. (2000). Health-related quality of life outcomes measures. Arch Phys Med Rehabil.

[94] Wing D., Nichols J.F., Parra M.T., Barkai H.S., Moran R.J. (2025). Digitally delivered, group-based exercise interventions for older adults: scoping review. J Med Internet Res.

[95] Peretti A., Amenta F., Tayebati S.K., Nittari G., Mahdi S.S. (2017). Telerehabilitation: review of the state-of-the-art and areas of application. JMIR Rehabil Assist Technol.

[96] Baroni M.P. (2023). The state of the art in telerehabilitation for musculoskeletal conditions. Arch Physiother.

[97] Skivington K. (2021). A new framework for developing and evaluating complex interventions: update of medical research council guidance. BMJ.

[98] Lewin S., Glenton C., Oxman A.D. (2009). Use of qualitative methods alongside randomised controlled trials of complex healthcare interventions: methodological study. BMJ.

