# Supplementary material for: Characterizing virtual community exercise programs for people with mobility limitations: a scoping review
Source: J Exerc Sci Fit. 2026 Apr 25;24(3):200477. doi: 10.1016/j.jesf.2026.200477 (PMC13193778; doi:10.1016/j.jesf.2026.200477)
Supplement: Multimedia component 1 [file mmc1.docx]

**Appendices** - **Characterizing virtual community exercise programs for people with mobility limitations: a scoping review**

Ovid MEDLINE search

| **#** | **Query** |
| --- | --- |
| 1 | telemedicine/ or remote consultation/ or distance counseling/ |
| 2 | ((comput* or distance or internet or phone or online or remote or tele* or video or virtual or web) adj2 (assess* or care or chat* or confer* or consult* or counsel* or deliver* or health* or interv* or medic* or rehabilitation or visit*)).tw,kf. |
| 3 | (teleassess* or telecare or teleconsult* or teledeliv* or telehealth* or teleinterv* or telemedic* or televisit* or teletherap* or tele exercise or tele-exercise or telerehabilitation or tele-rehabilitation or eConsult* or e-consult* or eHealth* or e-Health* or einterv* or e-interv* or etherap* or e-therap* or mHealth* or m-Health* or mobile health*).tw,kf. |
| 4 | (facetime* or store-and-forward* or store-forward* or skype* or video* or zoom or webbased or web-based).tw,kf. |
| 5 | exercise movement techniques/ or dance therapy/ or tai ji/ or yoga/ or exercise therapy/ or exp qigong/ or physical fitness/ or exp circuit-based exercise/ or exercise/ or cool-down exercise/ or muscle stretching exercises/ or physical conditioning, human/ or warm-up exercise/ |
| 6 | ((motor or movement* or task* or skill* or performance) adj3 (repetit* or repeat* or train* or re?train* or learn* or re?learn* or practice* or practis* or rehears* or rehers*)).tw,kf. |
| 7 | ((motor or movement* or task* or skill* or performance) adj3 (schedule* or intervention or program* or regim* or protocol*)).tw,kf. |
| 8 | (functional adj3 (task* or movement)).tw,kf. |
| 9 | (athletic performance or qi gong* or qigong* or tai ji or tai chi or yoga or pilates or dance therapy or physical activity or physical exercise program or aerobic exercise or anaerobic exercise or balance training or flexibility or stretch* or muscle stretching exercise* or strength* training or strength* exercise or resistance training or resistance exercise or physical fitness or physical conditioning or walking exercise or warmup exercise or warm up exercise or cooldown exercise or cool down exercise or circuit training or circuit class or task oriented training or task oriented exercise or repetitive task or task related training or task related exercise or task-specific or circuit based exercise or dynamic exercise or endurance training or exercise intensity or exergaming or leg exercise or low intensity exercise or moderate intensity exercise or muscle exercise or squatting exercise or static exercise or fitness).tw,kf. |
| 10 | dependent ambulation/ or walking speed/ or gait disorders, neurologic/ or gait apraxia/ or gait ataxia/ |
| 11 | paralysis/ or hemiplegia/ or paresis/ or paraparesis/ |
| 12 | ((gait or mobilit* or walk* or balance) adj2 (disorder* or problem or limitation* disabilit* or difficult* or ataxia or apraxia)).tw,kf. |
| 13 | cerebrovascular disorders/ or stroke/ or brain infarction/ or hemorrhagic stroke/ or ischemic stroke/ or brain diseases/ or basal ganglia cerebrovascular disease/ or brain ischemia/ or carotid artery diseases/ or cerebral small vessel diseases/ or cerebrovascular trauma/ or intracranial arterial diseases/ or intracranial arteriovenous malformations/ or "intracranial embolism and thrombosis"/ or intracranial hemorrhages/ or vascular diseases/ or brain diseases/ or brain injuries/ or brain hemorrhage, traumatic/ or brain injuries, diffuse/ or brain injuries, traumatic/ or brain injury, chronic/ or craniocerebral trauma/ |
| 14 | (stroke or poststroke or post stroke or cerebrovasc* or brain vasc* or cerebral vasc* or cva* or apoplex* or sah).tw,kf. |
| 15 | ((brain* or cerebr* or cerebell* or intracran* or intracerebral) adj4 (isch?emi* or infarct* or thrombo* or emboli* or occlus*)).tw,kf. |
| 16 | ((brain* or cerebr* or cerebell* or intracerebral or intracranial or subarachnoid) adj4 (haemorrhage* or hemorrhage* or haematoma* or hematoma* or bleed*)).tw,kf. |
| 17 | parkinsonian disorders/ or parkinson disease/ |
| 18 | (parkinson* adj2 (disease or disorders)).tw,kf. |
| 19 | ((traumatic or acquired) adj2 brain injury).tw,kf. |
| 20 | spinal cord diseases/ or amyotrophic lateral sclerosis/ or muscular atrophy, spinal/ or myelitis/ or pneumorrhachis/ or spinal cord compression/ or spinal cord injuries/ or spinal cord neoplasms/ or spinal cord vascular diseases/ or spinocerebellar degenerations/ or stiff-person syndrome/ or subacute combined degeneration/ or syringomyelia/ or tabes dorsalis/ |
| 21 | (spinal cord adj2 (injur* or diseas*)).tw,kf. |
| 22 | demyelinating autoimmune diseases, cns/ or multiple sclerosis/ or multiple sclerosis, chronic progressive/ or multiple sclerosis, relapsing-remitting/ |
| 23 | cancer survivors/ |
| 24 | arthritis/ or arthritis, infectious/ or arthritis, juvenile/ or arthritis, psoriatic/ or arthritis, rheumatoid/ or chondrocalcinosis/ or gout/ or osteoarthritis/ or periarthritis/ or rheumatic fever/ or sacroiliitis/ or spondylarthritis/ |
| 25 | frailty/ or frail elderly/ |
| 26 | cerebral palsy/ |
| 27 | neurodegenerative diseases/ or chronic traumatic encephalopathy/ or motor neuron disease/ or postpoliomyelitis syndrome/ |
| 28 | cerebellar diseases/ or cerebellar ataxia/ or cerebellar neoplasms/ or spinocerebellar degenerations/ or bulbar palsy, progressive/ or muscular atrophy, spinal/ or neuromuscular diseases/ |
| 29 | amputation, surgical/ or disarticulation/ or hemipelvectomy/ or disabled persons/ or amputees/ |
| 30 | (hemipar* or hemipleg* or paresis or paraparesis or tbi or pd or ms or scd or sci or cancer or arthritis or osteoarthritis or arthrosis or oa or arthritis or osteoarthritis or arthrosis or psoriatic arthritis rheumatoid arthritis or chondrocalcinosis or gout or periarthritis or rheumatic fever or sacroiliitis or spondylarthritis or pre-frail* or frail* cerebral palsy or neurodegenerative disease* or cerebellar disease* or neurodegenerative disease* or motor neuron disease* or amyotrophic lateral sclerosis or bulbar palsy or muscular atrophy* or neuromuscular disease* or neuromuscular disorder* or amputee or amputat* or disabled or muscular atrophy or myelitis or pneumorrhachis or spinal cord compression or spinal cord injuries or spinal cord neoplasms or spinal cord vascular diseases or spinocerebellar degenerations or stiff-person syndrome or subacute combined degeneration or syringomyelia or tabes dorsalis or demyelinating autoimmune diseases or multiple sclerosis chronic progressive or multiple sclerosis relapsing-remitting or postpoliomyelitis or disarticulation or hemipelvectomy).tw,kf. |
| 31 | or/1-4 |
| 32 | or/5-9 |
| 33 | or/10-30 |
| 34 | 31 and 32 and 33 |

**Table A.** Study methodology and exercise program characteristics of included studies (n=36)

| **Author (Year)**  **Country**  **Study design** | **Participants (sample size)** | **Structure of exercise programs** | **Content of exercise programs** | **Exercise program provider information** |
| --- | --- | --- | --- | --- |
| Adamson (2025)  USA  2-group pilot RCT | People with multiple sclerosis (n=22)  Mean age: 50.6 years  Female/Male: 16/6 | 60 min/class  2 classes/week  12 weeks  10-11 people/class  Remote and synchronous  Attendance: 55.68% (SD = 32.08) (including participants who dropped out); 69.79% (SD = 21.92) (excluding participants who dropped out) | Seated Pilates  *Control:* Seated Pilates+4 (4-quadrant stabilization exercises during the first six sessions) | Two certified Pilates instructors, including a peer with multiple sclerosis, combining clinical expertise and lived experience to ensure the intervention was inclusive and appropriate for participants across the spectrum of multiple sclerosis impairment. |
| Andonian (2024)  USA  2-group RCT | People with rheumatoid arthritis (n=20)  Mean age: 66.7 years  Female: 16 | Resistance training not specified. Aerobic training: recommended 150 min/week  1 class/week  16 weeks  people/class NR  Remote and synchronous  Attendance: 84.2% (17.1) resistance training component | SWET (Supervised Weight loss and Exercise Training): Aerobic training, resistance training, hypocaloric diet  *Control:* CHAT (Counseling for Healthy Activity and diet with Telehealth): diet and physical activity counseling | Exercise physiologist and registered dietitian |
| Aviram (2024)  Israel  3-group non-randomized trial | Cerebral palsy (n=40)  Mean age: 31 years  Female/Male: 26/14 | 60 min/class  2 classes/week  12 weeks  people/class NR  Remote, some classes asynchronous  Attendance: NR | Circuit resistance training  *Control:* in-person aerobic and resistance training | Coaches and physiotherapists under the supervision of a physiotherapist as well as a physical activity and sports specialist |
| Baehr (2023)  USA  Single group pre-post mixed methods study | People with spinal cord injury (n=11)  Mean age: 49.5 years  Female/Male: 5/6 | 45 min/class  2 classes/week  8 weeks  11 people/class  Remote, some classes asynchronous  Attendance: 62.5% | “Movement Connection”: strength training, aerobic training, mindfulness | Physiotherapist, co-led by an individual with spinal cord injury, providing lived experience feedback to the lead instructor and participants while demonstrating exercises throughout each session |
| Baehr (2024)  USA  Single group pre-post study | People with spinal cord injury (n=22)  Mean age: 46.8 years  Female/Male: 12/10 | 60 min/class  2 classes/week  8 weeks  22 people/class  Remote and synchronous  Attendance: NR | TEEMS (Tele-Exercise for Engaging with Movement and Self-Management):  strength training, aerobic training, mindfulness | Physiotherapist and community partner living with spinal cord injury |
| Callahan (2023)  USA  Single group pre-post study | People with traumatic brain injury (n=411)  Mean age: 44.9 years  Female/Male/Genderqueer: 335/68/5 | 45-75 min/class  2 classes/week  6 weeks  ≤13 people/class  Remote, some classes asynchronous  Attendance: NR | Yoga, meditation, group discussion  Asynchronous component: 4 prerecorded mindfulness tools sent to participants via e-mail | Trained facilitators and co-facilitators |
| Charlton (2023)  Canada  2-group pilot RCT | People with osteoarthritis (n=20)  Mean age: 67.4  Female/Male: 17/3 | 15-20 min/class  5 classes/week  6 weeks  1 person/class  Remote and synchronous  Attendance: 100% | Task-oriented training  *Control:* waitlist | Kinesiologist |
| Deepa (2023)  India  2-group RCT | People with Parkinson’s disease (n=44)  Mean age: 52.8 years  Women/Men: 44/0 | 60 min/class  4 classes/week  6 weeks  ≤3 people/class  Remote, some classes asynchronous  Attendance: NR | Prerecorded aerobic, balance and strength training shared on the online session and performed with the provider’s verbal instructions  *Control:* Nordic walking | Physiotherapist |
| Domingos (2022)  Portugal  Single group pre-post mixed methods study | People with Parkinson’s disease (n=15)  Mean age: 69.4 years  Female/Male: 9/6 | 60 min/class  2 classes/week  16 weeks  People/class NR  Remote and synchronous  Attendance: 81% | Task-oriented training combined with cognitive exercises | Physiotherapist |
| Donesky (2017)  USA  2-group non-randomized trial | People with both chronic obstructive pulmonary disease and heart failure (n=15)  Mean age: 71.7 years  Female/Male: 10/5 | 60 min/class  2 classes/week  8 weeks  7 people/class  Remote and synchronous  Attendance: 90% | Yoga  *Control*: attention control received educational materials (problems sleeping, elder abuse, flu vaccinations, accessing alternative therapies, medication information, depression, and a low sodium diet) | Certified yoga instructor who is also a physiotherapy assistant |
| Ekmekyapar Firat (2023)  Turkey  Single group pre-post study | People with Parkinson’s disease (n=15)  Mean age: 63.1 years  Female/Male: 6/9 | 60 min/class  4 classes/week  4 weeks  1 person/class  Remote, some classes asynchronous  Attendance: NR | LSVT-BIG (Lee Silverman Voice Therapy): large-amplitude whole-body movements to improve speed and function, requiring sustained attention and mental focus | Physiotherapist |
| Eldemir (2023)  Turkey  2-group RCT | People with multiple sclerosis (n=30)  Mean age: 39.7 years  Female/Male: 28/2 | 60 min/class  3 classes/week  6 weeks  1 person/class  Remote and synchronous  Attendance: 97.34% | Flexibility training, Pilates  *Control:* waitlist | Physiotherapist and certified Pilates instructor |
| Fishel (2024)  USA  2-group RCT | People with Parkinson’s disease (n=20)  Mean age: 68.8 years  Female/Male: 11/9 | 60 min/class  2 classes/week  8 weeks  10 people/class  Remote and synchronous  Attendance: group exercise: 99.4%; control group: 76.3% | Group exercise: Balance, aerobic and strength training  *Control:* Balance, aerobic and strength training delivered individually | Physiotherapist |
| Gagnon (2023)  Canada  Single group pre-post study | People with stroke (n=9)  Mean age: 60 years  Female/Male: 6/NR | 60 min/class  2 classes/week  12 weeks  3 people/class  Remote and synchronous  Attendance: NR | Balance, flexibility, and strength training | Kinesiologist |
| Galloway (2023)  Australia  4-group phase I modified 3+3 dose-escalation study | People with stroke (n=20)  Mean age: 62 years  Female/Male: NR/12 | 10-25 min/class (varied by cohort)  3 classes/week  8 weeks  People/class NR  Remote and synchronous  Attendance: 98.8% | Strength training  *Control:* same intervention different doses | Exercise scientist or a physiotherapist |
| Gomes Costa (2023)  Brazil  Longitudinal observation study | People with spinal cord injury (n=20)  Mean age: 36  Women/Men: 12/8 | min/class NR  3 classes/week  7 months  People/class NR  Remote and synchronous  Attendance: 45.1% | Strength training | Member of rehabilitation program with expertise in physical activity prescription for individuals with spinal cord injury |
| Ha (2024)  Korea  Single group pre-post study | People with Parkinson’s disease (n=56)  Mean age: 65.8 years  Female/Male: 34/20 | 40 min/class  2 classes/week  12 weeks  People/class NR  Remote and synchronous  Attendance: 60% | Aerobic and flexibility training | One registered nurse and one sports rehabilitation expert, who were both licensed yoga and Pilates instructors specializing in rehabilitative exercises for patients with movement disorders |
| Han (2022)  Korea  2-group non-randomized trial | Physically disabled people using wheelchairs (n=22)  Mean age: 49.4 years  Female/Male: 15/7 | 60 min/class  2 classes/week  6 weeks  People/class NR  Remote and synchronous  Attendance: NR | Aerobic, flexibility, strength training  *Control:* a YouTube link video of exercise for people with physical disabilities | Not reported |
| James-Palmer (2022)  USA, UK, Canada  Single group pre-post study | People with Parkinson’s disease (n=16)  Mean age: 63.1 years  Female/Male: 10/6 | 30 min/class  2 classes/week  6 weeks  People/class NR  Remote and synchronous  Attendance: 92.8% | Yoga | Physiotherapist who was a certified yoga instructor |
| Kannan (2024)  USA  2-group feasibility RCT | Frail older adults (n=27)  Mean age: 71.6 years  Female/Male: 17/10 | 90 min/class  3 classes/week  6 weeks  1 person/class  Remote and synchronous  Attendance: NR | MOB (Matter of Balance): group balance and strength training  *Control:* CogXergaming (aerobic, balance training and cognitive-motor function) | Researcher |
| Kaya Aytutuldu (2024)  Turkey  2-group RCT | People with Parkinson’s disease (n=34)  Mean age: 59.8 years  Female/Male: 8/24 | 60 min/class  4 classes/week  4 weeks  1 person/class  Remote and synchronous  Attendance: NR | LSVT-BIG (Lee Silverman Voice Therapy): large-amplitude whole-body movements to improve speed and function, requiring sustained attention and mental focus  *Control:* Task-oriented | Physiotherapist |
| Lai (2020)  USA  2-group pilot RCT mixed methods study | People with Parkinson’s disease (n=20)  Mean age: 67.1 years  Female/Male: 6/14 | 60 (week 1) to 165 min/class (week 8)  3 classes/week  8 weeks  1 person/class  First class in-person followed by remote and synchronous  Attendance: 99.2% (experimental group), 63.3% (control) | Aerobic and strength training  *Control:* self-regulated exercise using the telehealth system | Doctoral student supervised by a physiotherapist |
| Law (2023)  Canada  Single group pre-post study | People with Parkinson’s disease (n=17)  Mean age: 71.7 years  Female/Male: NR | 60 min/class  3 classes/week  12 weeks  17 people/class  Remote and synchronous  Attendance: NR | Tai chi | Tai Chi instructors with more than 4 years of experience |
| Li (2021)  USA  2-group pilot RCT | Older adults with mild cognitive impairment (n=30)  Mean age: 76.1 years  Women/Men: 21/9 | 60 min/class  2 classes/week  24 weeks  4 to 8 people/class  Remote and synchronous  Attendance: 79.2% (tai chi), 78.8% (control) | Dual-task Tai chi (interactive cognitive-physical exercises)  *Control:* stretching exercises | Not reported |
| Li (2023)  USA  3-group RCT | Older adults with mild cognitive impairment (n=318)  Mean age: 75.9 years  Female/Male: 212/106 | 60 min/class  2 classes/week  24 weeks  People/class NR  Remote, some classes asynchronous  Attendance: 80.63% (cognitively  enhanced tai chi), 79.5% (standard tai chi), and 79.7% (stretching) | Cognitively Enhanced Tai chi  *Controls:* standard tai chi, and stretch training | Not reported |
| Najafi (2023)  Malaysia and Iran  3-group RCT | People with multiple sclerosis (n=82)  Mean age: 40.8 years  Female/Male: 65/17 | 60 min/class  3 classes/week  8 weeks  People/class NR  Remote and synchronous  Attendance: maximum was 100% in both groups, while the minimum rate was 91% | Pilates  *Controls:* Yoga, and no intervention | Certified professional trainer with more than 10 years of experience, and a yoga instructor, with 12 years of experience working with PwMS |
| Najafi (2023)  Malaysia and Iran  3-group RCT | People with multiple sclerosis (n=45)  Mean age: 38 years  Female/Male: 45/0 | 60 min/class  3 classes/week  8 weeks  People/class NR  Remote and synchronous  Attendance: NR | Pilates  *Controls:* Yoga, and no intervention | Certified yoga instructor for Hatha yoga (12 years of experience with people with multiple sclerosis) and Pilates professional trainer for Tele-Pilates |
| Park (2023)  USA  2-group RCT | Older adults at risk for Alzheimer's disease and related dementias (n=32)  Mean age: 71 years  Female/Male: 30/2 | 45 min/class  2 classes/week  12 weeks  People/class NR  Remote and synchronous  Attendance: NR | Chair yoga  *Control:* computer brain games | Certified yoga instructor who had online yoga teaching experience with older adults |
| Park (2024)  Canada  Single group pre-post study | People with stroke (n=32)  Mean age: 62.5 years  Female/Male: 7/NR | 60-90 min/class  2 classes/week  4 weeks  2 people/class  Remote and synchronous  Attendance: NR | TRAIL (Telerehabilitation with Aims to Improve  Lower Extremity Recovery Poststroke): task-oriented training | Physiotherapists, who completed a 3-hour training specific for the program  Group format |
| Patel (2022)  USA  Single group pre-post study | Older adults with knee osteoarthritis (n=15)  Mean age: 71.8 years  Women/Men: 14/NR | 60 min/class  3 classes/week  12 weeks  People/class NR  Remote and synchronous  Attendance: 91.4% | Aerobic, balance, flexibility, and strength training | Experienced EnhanceFitness certified instructor. Assistant helped the instructor and participants trouble shoot any technical challenges |
| Pinto (2023)  Brazil  Single group pre-post study | People with Parkinson’s disease (n=26)  Mean age: 69 years  Female/Male: 23/3 | 60 min/class  2 classes/week  8 weeks  26 people/class  Remote and synchronous  Attendance: 87.5% | Online dance | Professional dancer and physiotherapist with extensive dance (25 years) and clinical (5 years) experience, supported by three research assistants for technical and safety needs. The instructor was trained in the Dance for PD® program and had prior in-person and online teaching experience. |
| Shah (2024)  USA  2-group feasibility RCT | People with knee osteoarthritis (n=40)  Mean age: 65.6 years  Female/Male: 35/5 | 120 min/class  1 class/week  8 weeks  6-15 people/class  Remote and synchronous  Attendance: 54% (mindful exercise), 68% (control) | Strength training and mindfulness-based stress reduction  *Control:* strength and balance training | Physiotherapist and a mindfulness instructor with ~20 years of experience |
| Tao (2022)  Canada  2-group RCT | Older adults with lower limb amputation (n=71)  Mean age: 64.9 years  Female/Male: 10/61 | 60-90 min/class  3 classes/week  8 weeks  3 people/class  First week in-person followed by 3 weeks remote and synchronous and 4 weeks asynchronous  Attendance: 89% (experimental group), 98% (control group) | Wii Fit balance board activities modified for balance training to improve walking capacity  *Control:* attention control used Big Brain Academy - Wii Degree with activities aimed at improving cognitive function | Trainers (research staff) |
| Tardelli (2023)  Brazil  Single group pre-post study | People with Parkinson’s disease (n=86)  Mean age: 66 years  Women/Men: 41/45 | 60 min/class  3 classes/week  10 months  12 people/class  Remote and synchronous  Attendance: 89% | SRTT (supervised, home-based, real-time videoconferencing telerehabilitation) | Physiotherapists |
| Thurston (2025)  Sweden  2-group feasibility RCT | People with stroke (n=114)  Mean age: 70.5 years  Female/Male: 73/NR | 60 min/class  2 classes/week (months 1 to 3)  1 class/week (months 4 to 6)  1 to 14 people/class  Remote, some classes asynchronous  Attendance: 76% | i-REBOUND: strength and aerobic training  *Control:* usual care | Physiotherapists |
| Wang (2022)  Canada  Single group pre-post study | Prefrail and frail older adults (n=30)  Mean age: 74 years  Female: 26/4 | 60 min/class  1 class/week  8 weeks  People/class NR  Remote and synchronous  Attendance: 84% | Yoga and behavior change support via autonomy-supportive instruction, social support, and journaling and reflection prompts | Exercise physiologist and a dietitian |

Abbreviations: RCT, randomized controlled trial; NR, not reported.

**Table B.** Pre- and post-intervention results for balance and mobility outcome measures in RCTs. Data are presented as mean (standard deviation), unless otherwise specified.

| **Population** | **Population**  **Timepoint** | **Outcome measure** | **Experimental group** | | | **Control group** | | |
| --- | --- | --- | --- | --- | --- | --- | --- | --- |
|  |  |  | **N** | **Pre** | **Post** | **N** | **Pre** | **Post** |
| Eldemir 2024 | People with multiple sclerosis  0-6 weeks | Berg Balance Scale | 15 | Median: 50 (IQR: 46–55) | 54 (52–56) | 15 | 55 (48–56) | 55 (50–56) |
|  |  | 6-Minute Walk Test (m) |  | 484.28 (64.50) | 517.93 (49.69) |  | 500.92 (83.17) | 483.76 (79.11) |
| Fishel 2024 | People with Parkinson’s disease  0-8 weeks | Five Times Sit-to-Stand | 10 | 9.00 (1.96) | 8.10 (1.45) | 10 | 10.75 (2.83) | 9.91 (2.89) |
|  |  | 6-Minute Walk Test |  | 445.55 (127.18) | 514.54 (61.27) |  | 467.07 (155.99) | 509.84 (148.28) |
|  |  | MiniBESTest |  | 24 (1.76) | 25 (1.33) |  | 23.3 (3.16) | 23.7 (2.45) |
| Kaya Aytutuldu 2024 | People with Parkinson’s disease  0-4 weeks | Mini-BESTest | 17 | 19.47 (2.74) | 24.24 (2.73) | 17 | 19.05 (3.61) | 22.49 (3.30) |
|  |  | Timed Up and Go Test |  | 9.03 (2.11) | 7.00 (1.92) |  | 10.31 (5.35) | 7.07 (1.93) |
|  |  | Activities-specific Balance Confidence |  | 57.88 (16.97) | 77.61 (14.73) |  | 59.84 (24.06) | 70.93 (22.32) |
| Li 2023 | Older adults with mild cognitive impairment  0-24 weeks | Timed Up & Go Test | 105 | 12.6 (standard error: 0.2) | 11.5 (0.2) | Standard Tai Ji: 107  Stretching: 106 | STJ: 12.7 (0.2)  Stretching: 12.5 (0.3) | STJ: 11.4 (0.2)  Stretching: 12.5 (0.2) |
|  |  | 4-Stage Balance Test |  | 2.6 (0.1) | 3.3 (0.1) |  | STJ: 2.6 (0.1)  Stretching: 2.6 (0.1) | STJ: 3.5 (0.1)  Stretching: 2.6 (0.1) |
|  |  | 30-second chair stands |  | 11.1 (0.2) | 11.8 (0.3) |  | STJ: 11.2 (0.2)  Stretching: 11.1 (0.2) | STJ: 12.6 (0.3)  Stretching: 11.1 (0.2) |
| Najafi 2023 | People with multiple sclerosis  0-8 weeks | Timed 25-Foot Walk | 15 | 8.36 (1.25) | 6.62 (1.21) | Tele-yoga: 15  Control: 15 | TY: 9.02 (2.04)  CG: 7.75 (1.32) | TY: 7.49 (2.02)  CG: 7.91 (1.44) |
| Park 2023 | Older adults at risk for Alzheimer's disease and related dementias  0-12 weeks | Timed Up & Go Test | 18 | 18.09 (8.71) | 17.89 (7.92) | 13 | 16.25 (10.60) | 16.25 (7.21) |
| Tao 2022 | Older adults with lower limb amputation  0-9 weeks | 2-Minute Walk Test | 38 | 125.6 (42.6) | 133.4 (46.2) | 33 | 126.5 (30.4) | 126.6 (31.0) |
|  |  | Short Physical Performance Battery |  | 15.2 (6.2) | 16.1 (7.0) |  | 15.2 (7.1) | 16.2 (7.4) |
|  |  | Four-Step Square Test |  | 14.6 (8.4) | 14.2 (9.8) |  | 12.9 (5.0) | 12.2 (3.7) |
|  |  | Activities-specific Balance Confidence |  | 83.3 (10.8) | 84.1 (11.1) |  | 82.7 (15.7) | 79.2 (19.7) |
